# The aconitate hydratase family from *Citrus*

**DOI:** 10.1186/1471-2229-10-222

**Published:** 2010-10-19

**Authors:** Javier Terol, Guillermo Soler, Manuel Talon, Manuel Cercos

**Affiliations:** 1 Centro de Genómica, Instituto Valenciano de Investigaciones Agrarias, Carretera Moncada - Náquera, Km. 4.5 Moncada (Valencia) E46113, Spain

## Abstract

**Background:**

Research on citrus fruit ripening has received considerable attention because of the importance of citrus fruits for the human diet. Organic acids are among the main determinants of taste and organoleptic quality of fruits and hence the control of fruit acidity loss has a strong economical relevance. In citrus, organic acids accumulate in the juice sac cells of developing fruits and are catabolized thereafter during ripening. Aconitase, that transforms citrate to isocitrate, is the first step of citric acid catabolism and a major component of the citrate utilization machinery. In this work, the citrus aconitase gene family was first characterized and a phylogenetic analysis was then carried out in order to understand the evolutionary history of this family in plants. Gene expression analyses of the citrus aconitase family were subsequently performed in several acidic and acidless genotypes to elucidate their involvement in acid homeostasis.

**Results:**

Analysis of 460,000 citrus ESTs, followed by sequencing of complete cDNA clones, identified in citrus 3 transcription units coding for putatively active aconitate hydratase proteins, named as *CcAco1*, *CcAco2 *and *CcAco3*. A phylogenetic study carried on the Aco family in 14 plant species, shows the presence of 5 Aco subfamilies, and that the ancestor of monocot and dicot species shared at least one *Aco *gene. Real-time RT-PCR expression analyses of the three aconitase citrus genes were performed in pulp tissues along fruit development in acidic and acidless citrus varieties such as mandarins, oranges and lemons. While *CcAco3 *expression was always low, *CcAco1 *and *CcAco2 *genes were generally induced during the rapid phase of fruit growth along with the maximum in acidity and the beginning of the acid reduction. Two exceptions to this general pattern were found: 1) Clemenules mandarin failed inducing *CcAco2 *although acid levels were rapidly reduced; and 2) the acidless "Sucreña" orange showed unusually high levels of expression of both aconitases, an observation correlating with the acidless phenotype. However, in the acidless "Dulce" lemon aconitase expression was normal suggesting that the acidless trait in this variety is not dependent upon aconitases.

**Conclusions:**

Phylogenetic studies showed the occurrence of five different subfamilies of aconitate hydratase in plants and sequence analyses indentified three active genes in citrus. The pattern of expression of two of these genes, *CcAco1 *and *CcAco2*, was normally associated with the timing of acid content reduction in most genotypes. Two exceptions to this general observation suggest the occurrence of additional regulatory steps of citrate homeostasis in citrus.

## Background

Citrus is one of the most important fruit crops in the world with a total production of 122 million tons in 2008 [1]. Citrus fruits are hesperidium berries with a very special organization composed of two morphologically distinct regions: the pericarp (peel) and the endocarp (pulp), which is the edible portion of the fruit and consists of segments, the ovarian locules, containing the juice vesicles. Growth and development of citrus fruit follows a sigmoid curve divided into three stages [2]. After an initial two months interval of slow growth mostly due to cell division (phase I), most of the fruit growth results from cell enlargement and water accumulation during the next 4 to 6 months (phase II). Finally, during phase III, growth is mostly arrested and fruits undergo a non-climacteric ripening process. Research on citrus fruit ripening has received considerable attention because of both the uniqueness of this physiological process and the importance of citrus fruits for the human diet as a supply of ascorbic acid, fibre and several phytochemicals with contrasted benefits for health [3].

Although color break, the pivotal metabolic event characterizing external ripening, takes place during phase III [4,5]; internal quality traits are acquired along both phases II and III [6]. During the first half of phase II, citrus fruits accumulate a considerable amount of organic acids in the vacuoles of the juice sac cells and these acids are progressively catabolized during the second half of phases II and III [7]. The physiological roles of organic acids in fruit cells are not fully understood, although it has been suggested that low pH could result in an enhanced sink strength, increasing carbohydrate uptake [8]. The characteristic decline in titrable acidity shown by many citrus fruits is due to the utilization of citric acid, the most abundant organic acid in citrus juice [9].

Mature citrus pulp contains a high percentage of water and many other different constituents, including acids and sugars among other determinants of taste and organoleptic quality [10]. Thus, the control of fruit acidity loss has a strong economical relevance since it is related to the consumer perception and hence constitutes a main constraint for the citrus industry. Recent advances in this field resulted in the identification of the main metabolic processes involved in citrate utilization [7,11,12] including a tonoplast citrate/H^+ ^symporter potentially involved in citrate efflux from the vacuole [13]. According to the current hypothesis, indirectly supported by proteomic data [14], citrate is released from the vacuole into the cytosol and then sequentially isomerized into isocitrate by cytosolic aconitase and then metabolized into 2-oxoglutarate by NADP^+^-isocitrate dehydrogenase. The utilization of 2-oxoglutarate involves transamination into glutamate and then either conversion into glutamine and further utilization for thiamine biosynthesis, or conversion into succinate through the gamma-aminobutirate shunt, eventually leading to carbohydrate synthesis. Alternatively, citrate may be converted to oxaloacetate and acetyl-CoA in a reaction catalyzed by ATP citrate lyase. However, since a decrease in ATP citrate lyase mRNA level during citrus fruit ripening has been reported [7] all steps involved in citrate utilization are initially dependent on its isomerization into isocitrate, the step controlled by aconitase.

Aconitase catalyzes the reversible isomerization of citrate to isocitrate via the intermediate product cis-aconitate. Two isoforms of aconitase have been detected in all eukaryotic cells: mitochondrial aconitase that is involved in the tricarboxylic acid cycle, and cytosolic aconitase that participates in several processes, such as cytosolic citrate metabolism [7,11,12] and the glyoxylate cycle [15,16]. It is also worth mentioning that aconitases are multifunctional proteins. In addition to the enzymatic activity, it has been shown, for instance, that the yeast mitochondrial aconitase is a component of the mitochondrial nucleoid and interacts with mitochondrial DNA [17], while the cytosolic aconitase has RNA binding activity related to iron homeostasis in animals and to resistance to oxidative stress in both animals and plants [18,19].

The aconitate hydratase family has been only described in detail in Arabidopsis [19,20]. However, the large number of vegetal genomes sequenced to completion in the last years allows performing extensive phylogenetic analysis in these species. Besides the ones from Arabidopsis, rice [21], poplar [22] and vitis [23], released several years ago, the genome sequences from many other plants are now available, and only during 2009 and the beginning of 2010 the genomes of corn [24], sorghum[25], soybean [26] and false purplebrome [27] have been completely sequenced. In this work, we have first characterized the aconitase gene family of citrus and performed, taking advantage of the availability of complete genome sequences, a phylogenetic analysis of the aconitate hydratase family in 14 species, which allowed an unprecedented view of the evolutionary history of this family in plants. In addition, the role of the citrus aconitase genes in the acid homeostasis has been investigated studying their expression in several acidic and acidless citrus genotypes.

## Results and discussion

### Molecular characterization of the Citrus aconitate hydratase genes

In order to identify cDNAs coding for ACO proteins, a BLASTX search was performed against 465,094 citrus ESTs available at the GenBank, using the *Arabidopsis thaliana *ACO proteins as queries. 179 ESTs produced significant similarity with the Arabidopsis ACO proteins, using an e value cut off of 1e-15. Assembly of the reads with GAP4 resulted in all the ESTs but one clustering into 3 contigs. The only singleton corresponded to a putative immature mRNA with an intron that prevented its assembly. The fact that all the ESTs clustered into 3 unigenes strongly supports that the aconitate hydratase family from citrus is composed, at least, of three transcription units. Three genes have been also reported in *A. thaliana*, the only plant species in which this family has been described in detail [19,20].

One cDNA clone representing each transcription unit was selected from a normalized full length library constructed from a variety of tissues and organs at different developmental stages and subjected to different abiotic stresses [28]. Clones IC0AAA40BB02RM1, IC0AAA5BE08RM1, and IC0AAA7DC07RM1 were sequenced to completion with a primer walk strategy, resulting in 3 sequences of 3218, 3331, and 3433 bp respectively, that were submitted to GenBank (Acc# FN552254, FN552255, and FN552256). Conceptual translation of the cDNA sequences showed that clones IC0AAA40BB02RM1, IC0AAA7DC07RM1, and IC0AAA5BE08RM1 contained ORFs coding for proteins of 900, 898, and 898 aa, respectively, named *Aco1*, *Aco2 *and *Aco3 *genes, based on their similarity with the Arabidopsis ones (see Table 1 for details). An aconitate hydratase gene from *Citrus limon *that was previously reported [GenBank:AF073507] was 99% identical to *Aco3 *from *C. clementina*.

**Table 1:** The Aconitate hydratase genes in *Citrus*

	cDNA clone	Acc N°	bp^a^	st^b^	end^b^	ORF^d^	aa^e^
***CcAco1***	IC0AAA40BB02RM1	FN552254	3218	103	2805	2700	900
***CcAco2***	IC0AAA7DC07RM1	FN552256	3433	520	3216	2694	898
***CcAco3***	IC0AAA5BE08RM1	FN552255	3331	443	3139	2694	898

^a ^mRNa length in bp

^b ^Start codon position

^c ^Stop codon position

^d ^ORF length in bp

^e ^Protein length in aa

**Figure 1:**
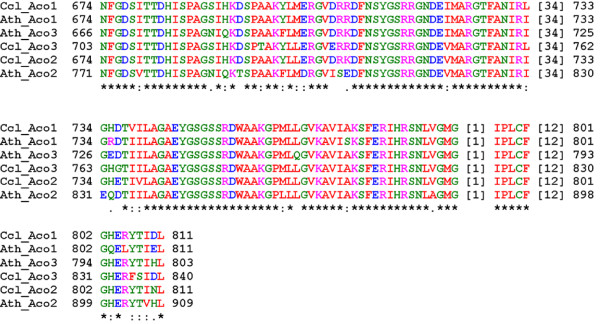
**Conservation of the swivel domain in Arabidopsis and Clementine ACO proteins**. A multiple alignment of the swivel domain of the aconitate hydratase proteins from Arabidopsis and *C. clementina *is shown. Asterisks show identical residues in all six sequences, colons indicate conservative changes, and points represent semi-conserved substitutions.

A multiple alignment of the Arabidopsis and Clementine proteins with ClustalX exhibited a high degree of conservation between the six proteins, with 627 identical residues, and 142 conservative changes, representing an overall similarity higher than 85%. Analysis of the citrus predicted proteins with PFAM [29] showed conservation of the catalytic and the swivel domains [20], suggesting that the citrus proteins are able to catalyze the interconversion of isocitrate and citrate via a cis-aconitate intermediate (Figure 1).

### Phylogenetic analysis of the aconitate hydratase family in plants

In order to study the evolutionary history of the aconitate hydratase family in plants, the complete genome sequences of the dicot species *Populus trichocarpa *[22], *Vitis vinifera *[23], *Carica papaya *[30], *Ricinus communis *[31], *Medicago truncatula *[32], and *Glycine max *[26]; the monocot species *Oryza sativa *[21], *Zea mays *[24], *Sorghum bicolor *[25], and in *Brachypodium distachyon *[27], and the moss *Physcomitrella patens *[33] were searched for *Aco *genes. The BLASTX search identified 3 *Aco *genes in all but 3 species, which is in agreement with the results from Arabidopsis and citrus. Soybean and poplar displayed 6 and 4 *Aco *genes, respectively, while only 2 transcription units were discovered in purple false brome. Table 2 shows the *Aco *ORFs obtained, indicating the species and their accession numbers.

**Table 2:** The Aconitate hydratase genes in plants

Species	ORF Acc. N°	Protein Acc. N°	Locus	Aco class	Associated ESTs
*A. thaliana*	NM_119749	NP_195308	At4g35830	Aco1	47
	NM_126589	NP_178634	At2g05710	Aco2	183
	NM_118831	NP_567763	At4g26970	Aco3	131
*C. clementina*	FN552254	CBE71056	-	Aco1	46
	FN552256	CBE71058	-	Aco2	93
	FN552255	CBE71057	-	Aco3	35
*C. papaya*	DS981532	-	-	Aco1	7
	DS981607	-	-	Aco2	1
	DS981526	-	-	Aco3	14
*M. truncatula*	CR932965_17	-	MT060719-3729.M00012	Aco1	61
	AC173287_15	-	MT060719-3496.M00013	Aco2	40
	AC144481_23	-	MT060719-2520.M00023	Aco3	2
*P. trichocarpa*	XM_002327692	XP_2327728	POPTRDRAFT_593790	Aco1	29
	XM_002331719	XP_2331755	POPTRDRAFT_585679	Aco1	4
	XM_002301587	XP_2301623	POPTRDRAFT_816803	Aco2	57
	XM_002321090	XP_2321126	POPTRDRAFT_246575	Aco2	5
*R. communis*	XM_002530589	XP_002530635	RCOM_0782740	Aco1	2
	XM_002524138	XP_002524184	RCOM_0487910	Aco2	12
	XM_002532518	XP_002532564	RCOM_0082520	Aco3	0
*V. vinifera*	XM_002263301	XP_2263337	LOC100242027	Aco1	29
	XM_002279224	XP_2279260	LOC100256776	Aco2	18
	XM_002278102	XP_2278138	LOC100253811	Aco3	38
*G. max*	BT095399		Glyma01g36750	Aco1	435
	AK286137	-	Glyma11g08550	Aco1	61
	-	-	Glyma06g46190	Aco3	38
	AK244974	-	Glyma12g10580	Aco3	25
	AK286541	-	Glyma12g32000	Aco3	55
	-	-	Glyma13g38480	Aco3	55
*O. sativa*	NM_001055433	NP_001048898.1	Loc_Os03g04410	Aco1	194
	AP005505	BAD05751	Loc_Os08g09200	Aco4	216
	NM_001063996	NP_001057461	Loc_Os06g19960	Aco5	0
*S. bicolor*	XM_002465856	XP_2465901	Sb01g047850	Aco1	17
	XM_002445129	XP_2445174	Sb07g005390	Aco4	75
	-	-	Sb06g000210	Aco5	38
	XM_002460720	XP_2460765	Sb02g034590	-	14
*B. distachyon*	-	-	Bradi1g75960	Aco1	3
	-	-	Bradi3g15050	Aco4	40
*Z. mays*	NM_001165757	NP_001159229	LOC100304315	Aco1	188
	NM_001143012	NP_001136484	LOC100216599	Aco4	301
	NM_001153959	NP_001147431	-	Aco5	128

**Figure 2:**
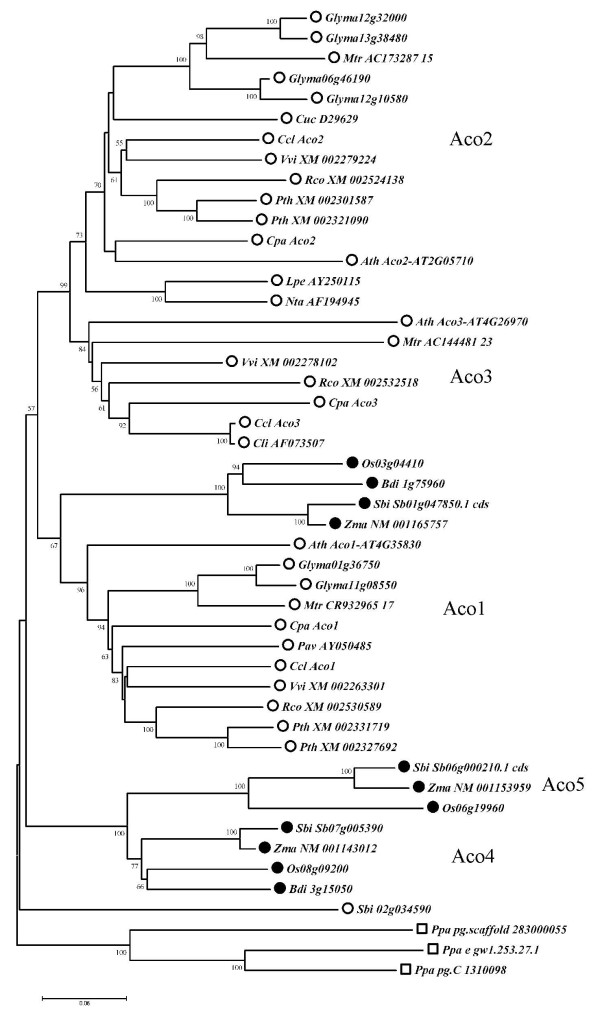
**Evolutionary relationships of the aconitate hydratase family in plants**. The evolutionary history was inferred using the Neighbor-Joining method. The optimal tree with the sum of branch length = 4.42716789 is shown. Only significant bootstrap values are shown next to the branches. The tree is drawn to scale, with branch lengths in the same units as those of the evolutionary distances used to infer the phylogenetic tree. There were a total of 3737 positions in the final dataset. The different Aco subfamilies are indicated with labels close to the corresponding branches. Empty circles indicate dicot species, black circles designate monocot ones, squares mark the bryophyte *P. patens*. Species names are abbreviated as: Ath (Arabidopsis), Bdi (false purplebrome), Ccl (Clementine), Cli (lemon), Cpa (papaya), Cuc (cucumber), Glyma (soybean), Lpe (tomato), Mtr (barrel medic), Nta (tobacco), Osa (rice), Pav (peach), Ppa (P. patens), Pth (poplar), Rco (castor oil), Sbi (sorghum), Vvi (grapevine), and Zma (corn).

A multiple alignment with 48 *Aco *sequences was carried out, and a phylogenetic analysis was performed as described in Methods. The tree obtained (Figure 2) shows six main clusters that include all but one *Aco *ORFs. As expected, sequences from *P. patens *group in a single cluster with the largest genetic distances respect to the other sequences, which reflects the evolutionary distance between Bryophyta and Angiosperm taxa. All the remaining sequences but Sbi_02g034590, group into 5 clusters, that have been named Aco1, Aco2, Aco3, Aco4, and Aco5, based on the Arabidopsis naming system.

Sequence Sbi_02g034590 from sorghum, annotated as a member of the aconitate hydratase family in the BLAST searches, is not included in the previous clusters. Genetic distances with respect to the other sequences are also in the same range than the ones obtained for *P. patens *suggesting that it should not be considered as part of this family.

Aco1 cluster is composed of sequences from both mono and dicot species, indicating that their ancestor was present in plants previously to the split of mono and dicotyledonous groups. Subclusters Aco2 and Aco3 only gather genes from dicot species, while Aco4-Aco5 clades are formed only by monocot ones. This fact suggests that these clusters appeared after the monocot and dicot split, and have diverged to give rise to new subfamilies. It is noteworthy that all the dicot species, except poplar and soybean, display 3 *Aco *genes, that are distributed into the Aco1, Aco2 and Aco3 clusters. The organization of the monocot subclusters was found to be similar. Analogous evolutionary patterns with sequences from mono and dicot species distributed among mono and polyphyletic clades have been also found for the polygalacturonase [34], lignin biosynthesis [35], or DOF [36] gene families.

The Aco family in poplar and soybean displays a different organization since both genomes lack *Aco3 *homologs, and their genes group in Aco1 and Aco2 clusters with very small genetic distances between them, suggesting the occurrence of recent duplication events. In fact, XM_002301587 and XM_002321090 are located on homologous genome blocks from chromosomes II and XIV, produced in a salicoid-specific genome-wide duplication event described in poplar that is still detectable over approximately 92% of the poplar genome [22]. The salicoid duplication has been also related to the fact that *Populus *has the largest number of major intrinsic proteins (MIPs) identified in any single plant species [37], with the duplication of several cinnamyl alcohol dehydrogenase (CAD) genes [38], or with the fact that the neutral invertases (NIs) gene family has 9 members in the *Arabidopsis *genome, 8 in rice, 9 in vitis, while 16 in poplar [39]. Similarly, the 6 Aco genes found in soybean might be originated by succeeding whole-genome duplications, one of them legume specific 13 MYA ago, and by an extraordinary level of gene retention occurred in this species, resulting in 75% of the soybean genes present as multiple copies [26].

In order to find how many of the predicted ORFs were transcriptionally active, a BLAST search against the GenBank EST database was performed with the species analyzed in the phylogenetic study. For each species, the predicted ORFs and the ESTs producing significant scores were assembled with GAP4 (see Methods), which resulted in contigs containing the ORF sequences and all the ESTs associated with each hypothetical gene (Table 2). Considering the large number of analyzed ESTs in several species (1.5 MM in Arabidopsis, 1.2 MM in rice, 260.000 in Medicago, etc), the difference in the number of ESTs found between the *Aco *genes may reflect the different expression levels of the genes in them.

It is noteworthy that in poplar, with more than 426.000 ESTs analyzed, only one predicted gene per cluster, XM_002327692 (27 ESTs) and XM_002301587 (33 ESTs), show significant levels of expression, while their pairs (XM_002331719, and XM_002321090) only show none and 2 associated ESTs. Chaudhary et al. (2009) described a similar expression asymmetry in the analysis of the carotenoid biosynthesis genes in rice, Arabidopsis and poplar [40]. The analysis of the polygalacturonase gene family in Arabidopsis and rice also showed that tandem duplicated regions had one relatively highly expressed gene while the rest had either low or no expression levels [34], Finally, unequal expression of homologous neutral invertase genes was observed by quantitative RT-PCR in vitis [39]. These changes in the level of expression of duplicated genes have been related with neofunctionalization after gene duplications [41].

### Expression of aconitase genes

To understand the physiological roles of *CcAco1*, *CcAco2 *and *CcAco3 *genes and their involvement in the control of fruit acidity levels, their expression in pulp tissues was determined by real-time RT-PCR along fruit development in selected varieties of mandarins, oranges and lemons. Total acidity was also determined in juice extracts to correlate fruit acidity with aconitase gene expression. In mandarins, analyses were performed on two varieties differing in fruit acidity: Clemenules (*Citrus clementina *Hort. ex Tan. cv. Clemenules) and Fortune (*Citrus clementina *Hort. ex. Tan. x *Citrus reticulata *Blanco). Three varieties of sweet orange (*Citrus sinensis *L. Osb.) were also used: an acidless orange (cv. Sucreña) and two normal acidic ones (cv. Comuna and cv. Valencia Late). The two lemon varieties (*Citrus limon *L. Burm.) studied were cv. Dulce, that is an acidless lemon and cv. Fino that shows normal acidity levels.

**Figure 3:**
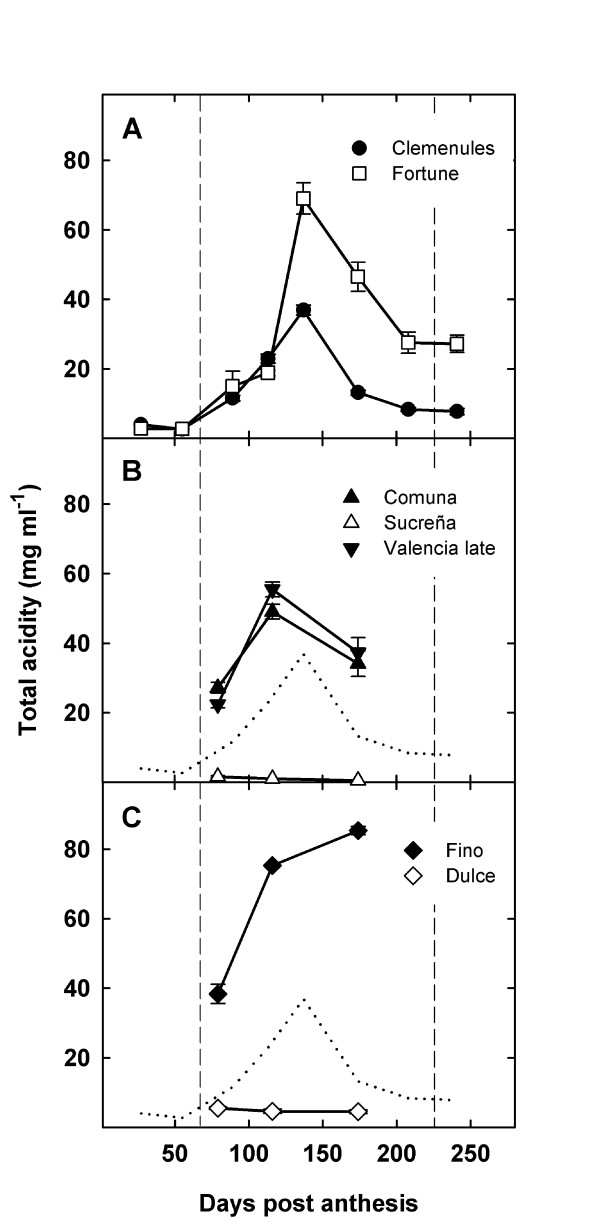
**Changes in acidity during fruit development**. Total titrable acidity in juice extracts of selected varieties of mandarins (A), oranges (B) and lemons (C) during pulp development. Clemenules acidity profile (dotted line) is included in panels B and C as a reference. Vertical dashed lines separate the three phases of citrus fruit development: cell division (phase I), cell expansion (phase II) and ripening (phase III).

In Clemenules mandarin, total acidity was low during phase I of fruit development (cell division stage), started to increase at the beginning of phase II (cell enlargement stage), reached the maximum levels around 140 days post anthesis and decreased thereafter during the second half of phase II and during phase III (ripening stage). Fortune mandarin acidity showed a similar profile, although acid content was significantly higher at the acidity peak (69.03 ± 4.53 mg/ml in Fortune versus 36.95 ± 1.40 mg/ml in Clemenules) and remained higher during the second half of phase II and in mature fruit (Figure 3A), when acid content was 7.74 ± 0.89 mg/ml in Clemenules fruits and 27.20 ± 2.54 mg/ml in Fortune fruits. Similarly, acidity in Comuna and Valencia Late orange varieties also showed a peak in the middle of phase II at 116 DPA, with acidity levels of 49.07 ± 2.13 mg/ml and 55.47 ± 2.13 mg/ml respectively (Figure 3B). Total acidity was extremely low in the acidless Sucreña orange and acidity at 116 DPA was 1.06 ± 0.05 mg/ml. In cultivar Fino, a normal acidity lemon, acid content also increased during the second half of phase II reaching levels much higher than that of mandarin and orange varieties (for example 85.37 ± 1.18 mg/ml at 174 DPA, Figure 3C). As above, in acidless Dulce lemon, no acidity increase was observed and acid levels remained low and slightly higher than those of Sucreña orange (4.61 ± 0.67 mg/ml at 116 DPA).

**Figure 4:**
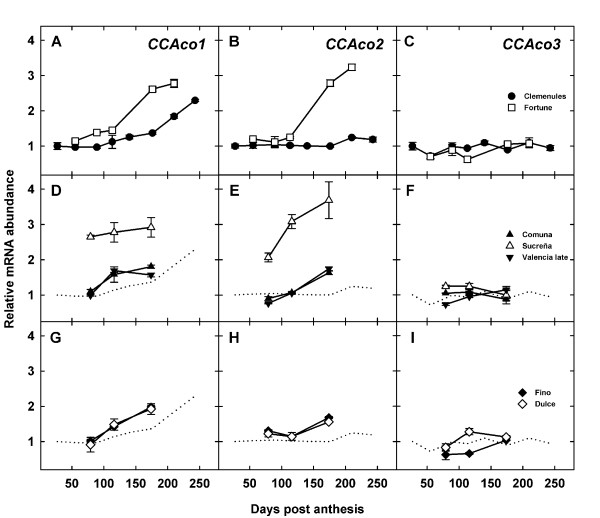
**Aconitase expression during fruit development**. Expression profiles of citrus aconitase genes *CCAco1 *(A, D, G), *CcAco2 *(B, E, H) and *CCAco3 *(C, F, I), during development of fruit flesh from selected varieties of mandarins (A, B, C), oranges (D, E, F) and lemons (G, H, I). Gene expression was determined by real-time RT-PCR. An expression value of 1 was arbitrarily assigned to the mRNA level of each gene in the Clemenules 27 DPA sample. Mean ratios of expression ± SE are presented. Clemenules expression profiles (dotted lines) are included in panels D, E, F, G, H and I as reference.

Quantitative real-time RT-PCR analyses (Figure 4) allowed transcript detection for *CcAco1*, *CcAco2 *and *CcAco3 *indicating that these sequences correspond to functional aconitase genes. *CcAco3 *was constitutively expressed although at low levels along all phases of fruit development in all citrus varieties used for this study (Figure 4C, F, I). Thus, this expression pattern suggests that *CcAco3 *would not be deeply involved in the acidity reduction process taking place in the cytosol during pulp ripening and that the CcAco3 protein might have a mitochondrial localization and therefore be involved in the tricarboxylic acid cycle taking place in the mitochondrial matrix. However, cytosolic and mitochondrial compartimentalizations are not mutually exclusive since evidences for the simultaneous localization of plant and yeast aconitase proteins in cytosol and mitochondria have been reported [42].

In general, increase of *CcAco1 *and *CcAco2 *gene expression started during the first middle part of phase II (Figure 4A, B). Thus, induction of these genes was coincident or slightly preceded the acidity peak while expression was high at the beginning and during the reduction in acid level. This suggests that *CcAco1 *and *CcAco2 *proteins are presumably related to the cytosolic aconitase activity metabolizing the citrate released from the vacuole during ripening [7,11]. Interestingly, Clemenules fruits did not show *CcAco2 *induction, an observation suggesting a possible mutation in the regulatory regions of this gene, since no alteration was detected in the coding sequence (data not shown). In spite of the lack of *CcAco2 *induction in Clemenules, acidity and *CcAco1 *and *CcAco2 *mRNA levels in Fortune were still higher than those in Clemenules. Thus, differences in citric acid content between these two varieties may be related to additional regulatory steps such as the activity of the membrane transporters mediating citrate efflux from the vacuole during ripening [13]. Although the mechanisms controlling citrate vacuolar compartmentalization in citrus are not fully understood, several regulatory points have been proposed for the transport of citrate anions across the tonoplast, including a thermodynamically favoured transport through rectifying anion channels [13] and an active ATP-driven transport mechanism [43]. In addition, Shimada et al [13] isolated a novel citrate-H^+ ^symporter induced during fruit acidity decrease in Washington Navel. The elucidation of the mechanisms involved in the control of vacuolar citrate compartmentalization may thus help to understand the differences in acidity found between these mandarin varieties.

The highest mRNA levels of *CcAco1 *and *CcAco2 *genes were found in the pulp of acidless Sucreña orange and these levels were already high at the onset of phase II, when the expression of these genes was still arrested in normal acidity oranges (Comuna and Valencia Late) mandarins and lemons. The overexpression of these genes when citric acid is being produced in the mitochondrial matrix, released to the cytosol and exported to the vacuole may be responsible for the acidless phenotype of the variety, since a higher aconitase activity would degrade citric acid, avoiding its vacuolar accumulation.

The comparison of Dulce, an acidless lemon variety with Fino, a normal acidity lemon, showed no significant differences in the expression of the putative cytosolic aconitase genes. This suggests that different mechanisms are responsible for the phenotypes of orange (Sucreña) and lemon (Dulce). Furthermore, in limes, a derived species from lemon, an explanation involving additional players has been previously proposed for the acidless trait of sweet lime [44]. Sadka et al. [11] reported that during the initial stages of fruit development a partial inhibition of the enzymatic activity of lemon mitochondrial aconitase allowed the production of high amounts of citric acid in the mitochondria during the initial stages of fruit development. This activity decrease was correlated with a previously reported increase in citramalate, a competitive inhibitor of aconitase activity [44] In acidless sweet lime, citramalate level was lower [44], suggesting that the acidless phenotype of this variety mostly related to citric acid production instead of citric acid removal. This possibility is compatible with the observation that in acidless Dulce lemon, expression of the genes involved in citrate removal was normal (Figure 4 G, H) and expression of one aconitase gene observed at 79 and specially at 116 DPA (Figure 4I) was slightly increased.

An additional finding of this work is that despite the strong accumulation of citric acid in lemons (Figure 3C), *CcAco2 *aconitase mRNA levels were similar to those found in oranges and mandarins (Figure 4G, H). This suggests that citric acid in ripening lemon fruit may not be accessible to the aconitase proteins and reinforces the above idea that regulatory differences in the citrus tonoplast transporters may be relevant to understand the diversity of patterns of citric accumulation and homeostasis in the several citrus fruits.

## Conclusions

Phylogenetic studies showed the occurrence of five different subfamilies of aconitate hydratase genes in plants that derived from one common ancestor of mono and dicots. The analysis of the sequence of the aconitase family in citrus showed that it is composed of three active genes. The pattern of expression of two of these genes, *CcAco1 *and *CcAco2*, was normally associated with the timing of citrate reduction in most genotypes including mandarins, oranges and lemons. In addition, two exceptions to this general observation suggest the occurrence of additional regulatory steps of citrate homeostasis in citrus.

## Methods

### Plant material

Parthenocarpic citrus fruits were harvested from adult trees grown in the field under normal cultural practices. The following acidic and acidless varieties and species were used: Clementina mandarin (*Citrus clementina *Hort. ex Tan.; cv. Clemenules), Fortune mandarin (*Citrus clementina *Hort. ex. Tan. x *Citrus reticulata *Blanco), three sweet orange varieties (*Citrus sinensis *L. Osb.; cv. Comuna, cv. Sucreña and cv. Valencia Late) and two lemon varieties, (*Citrus limon *L. Burm.; cv. Fino and cv. Dulce). Sucreña orange and Dulce lemon are characterized by a very low level of acid contain and therefore are defined as acidless varieties. Fruits were selected by uniformity of size and appearance, and absence of abiotic and biotic stress symptoms. Homogeneous fruits were peeled and flavedo (exocarp) and albedo (mesocarp) discarded. The remaining tissue, mainly consisting of juice vesicles (endocarp), the segments with their membranes and the vascular bundles, was frozen under liquid nitrogen and stored at -80°C until used.

Two samples of Clemenules were taken during phase I (cell division stage) on May 27, at 27 days post anthesis (DPA), and June 24, at 55 DPA. Five samples were harvested during phase II (cell expansion stage), two before total acidity peak (July 28, 89 DPA, and August 21, 113 DPA), one at maximum acidity (September 17, 140 DPA) and two after the peak of maximum acidity (October 23, 176 DPA and November 26, 210 DPA). The last sample was harvested during phase III (ripening) at the fully ripe stage (December 29, 243 DPA), when acidity content was again low. Samples of Fortune mandarin were harvested at the same times. Fruits of the sweet orange varieties Comuna, Sucreña and Valencia Late, and from the two lemon varieties, Fino and Dulce were harvested during phase II, around the maximum acidity peak of Clemenules, at 79, 116 and 174 DPA. To exclude differences in gene expression due to environmental factors, two sets of samples were taken during the years 2005 and 2008. The data reported here were obtained with the 2005 samples, and similar expression patterns were observed with the 2008 samples (data not shown).

### cDNA Sequencing

Plasmid DNA was extracted from liquid culture (LB plus Amp) with the FastPlasmid Mini Kit (Eppendorf), following manufacturer's instructions. cDNA clones were sequenced to completion by primer walk using the primers shown in additional file 1 Table S1. Sequencing reactions were performed utilizing the ABI Big Dye Terminator Cycle Sequence Ready Reaction as described by manufacturer in an ABI 3730 automatic sequencer. Reads were assembled with the software GAP4 from the Staden package [45], and a consensus sequence was obtained for each transcription unit.

### Phylogenetic analysis

The BLASTX tool was used to discover Aco genes in Populus trichocarpa [22], Vitis vinifera [23], Carica papaya [30], Ricinus communis [31], Medicago truncatula [32], Glycine max [26], Oryza sativa [21], Zea mays [24], Sorghum bicolor [25], and Brachypodium distachyon [27]. The moss Physcomitrella patens [33], was also analyzed as a representative of more primitive taxonomic groups. Additionally, the ORFs of the plant aconitate hydratase proteins from Citrus limon [Genbank:AF073507], Cucurbita sp [GenBank:D29629], Lycopersicon pennelli [GenBank:AY250115], Nicotiana tabacum [GenBank:AF194945], and Prunus avium [Genbank:AY050485], previously described and available at GenBank, were also included in this study.

Although the genome sequence of papaya has been published [30], only DNA sequences produced by the whole genome shotgun (WGS) are accessible at the GenBank. In order to obtain the aconitate hydratase genes from papaya, a BLASTX search was performed against the WGS scaffolds available at the public database. Three scaffolds yielding significant homology were obtained, and ORF prediction was performed with GeneScan [46], followed by manual curation. The 3 ORFs obtained were named *Aco1*, *Aco2 *and *Aco3 *based on their similarity with Arabidopsis, and were also included in the phylogenetic analysis.

A multiple alignment with the 48 predicted Aco ORFs was carried out with Clustal_X [47]. Phylogenetic analyses were conducted in MEGA4 [48]. Genetic distances were calculated with the Maximum Composite Likelihood method [49] and all positions containing alignment gaps and missing data were eliminated only in pairwise sequence comparisons (Pairwise deletion option). There were a total of 3.595 positions in the final dataset. The phylogenetic tree was constructed with the Neighbor-Joining method [50]. The percentage of replicate trees in which the associated taxa clustered together was calculated with the bootstrap test with 1000 replicates [51]. Similar analyses were performed with the Minimum Evolution [52] and Maximum Likelihood methods [53], and the trees obtained confirmed the clustering obtained with the Neighbor-Joining method (data not shown).

BLASTN [54] search tool was used to identify ESTs associated with the Aco ORFs against the EST section of the GenBank [55]. For each species, the ESTs obtained were assembled with the ORF sequences using GAP4 from the Staden package [45], which allowed counting of the number of identical ESTs associated with an ORF.

### Fruit acidity determination

Fruit acidity was determined by titration of 5 ml of fresh juice extract with 0.1 M NaOH, using phenolphtalein as indicator. Total acidity was used as an indirect measurement of citric acid concentration, the dominant acidic compound in Citrus fruit juice and major responsible of fruit acidity [9].

### RNA extraction and real-time RT-PCR

Total RNA was isolated from frozen tissue using the RNeasy Plant Mini Kit (Qiagen) and treated with RNase-free DNase (Qiagen) according to the manufacturer's instructions. UV light absorption spectrophotometry and agarose gel electrophoresis were performed to test RNA quality as described by Sambrook et al. [56] and RNA concentration was accurately determined by a fluorometric assay with the RiboGreen dye (Molecular Probes) following the manufacturer's instructions. Quantitative real-time RT-PCR was performed with a LightCycler 2.0 Instrument (Roche) equipped with LightCycler Software version 4.0 as described by [7]. One-step RT-PCR was carried out with 25 ng total RNA adding 2.5 units of MultiScribe Reverse Transcriptase (Applied Biosystems), 1 unit RNase inhibitor (Applied Biosystems), 2 μl of LC FastStart DNA MasterPLUS SYBR Green I (Roche) and 2.5 pmol of each oligonucleotide in a total volume of 10 μl. Incubations were carried out at 48°C for 30 min, 95°C for 10 min followed by 40 cycles at 95°C for 20 s, annealing temperature (55°C for *CcAco1 *and *CcAco3 *and 58°C for *CcAco2*) for 10 s and 72°C for 15 s. Fluorescent intensity data were acquired during the 72°C extension step. Gene-specific primers (Table 3) were designed using the Primer Express software (Applied Biosystems). Specificity of the amplification reactions was assessed by postamplification dissociation curves and by sequencing the reaction products. To transform fluorescent intensity measurements into relative mRNA levels, a 10-fold dilution series of a RNA sample was used as standard curve. Reproducible data were obtained after normalization to total RNA amounts [57] since previous work in our lab indicated that other normalization methods rendered irreproducible results [4]. For proper comparison, data were re-scaled so that an induction value of 1-fold was arbitrarily assigned to the 27 DPA Clemenules sample. Each sample was analyzed in triplicate and mean ratios ± standard errors were calculated.

**Table 3:** Oligonucleotides used as primers for real-time RT-PCR

Gene	Primer orientation	Primer sequence	Amplicon position
*CcAco1*	Forward	5'-GGCAAGTCATTCACATGCGTT-3'	2692-2852
	Reverse	5'-TGAAGAAGTAGACCCCGGTTGA-3'	
*CcAco2*	Forward	5'-GGCAATGATGAAGTGATGGCT-3'	2671-2771
	Reverse	5'-GTTGGAACATGGACCGTCTTT-3'	
*CcAco3*	Forward	5'-TGCAGCAATGAGGTACAAGGC-3'	2719-2834
	Reverse	5'-TCACACCCAGAAGCATTGGAC-3'	

## Authors' contributions

JT carried out characterization of the citrus Aco genes, the phylogenetic analysis in plants and drafted the manuscript. GS performed the RT-PCR analysis. MT and MC coordinated the project and drafted the manuscript. All authors read and approved the final manuscript.

## Supplementary Material

Additional file 1 **Primers used in cDNA sequencing**. This excel file show the oligos used in the primer walk performed to sequence the 3 cDNA clones from *C. Clementina*. Click here for file
